# Analysis on the pharyngeal microbiota in patients with laryngopharyngeal reflux disease^☆^

**DOI:** 10.1016/j.bjorl.2023.101331

**Published:** 2023-09-18

**Authors:** Jintang Zhang, Xiaofan Fan

**Affiliations:** The Fourth Affiliated Hospital, Zhejiang University School of Medicine, Department of Otolaryngology, Yiwu, China

**Keywords:** Laryngopharyngeal reflux disease, Microbiome, Receiver operating characteristic curve, Outcomes

## Abstract

**Objective(s):**

In this study, the laryngopharynx microbiome alterations were characterized after proton pump inhibitor treatment in patients with Laryngopharyngeal Reflux Disease (LPRD) and healthy people. The potential outcome-predictive biomarker was explored.

**Methods:**

Patients with LPRD and healthy controls were enrolled. The composition of their laryngopharynx microbiota was analyzed both by traditional plate count of the main bacterial groups and PCR amplification followed by denaturing gradient gel electrophoresis. Shannon-Wiener index and evenness index based on Dice index were used to assess the bacterial diversity. Droplet digital PCR was used to determine the total bacterial RNA and relative abundance of *Klebsiella oxytoca*. Receiver operating characteristic curve was plotted to explore the potential of *Klebsiella oxytoca* as an outcome-predictive biomarker.

**Results:**

A total of 29 LPRD cases and 28 healthy subjects were enrolled. The composition of the laryngopharynx microbiota was almost similar, except *Klebsiella oxytoca*. The cluster analysis showed that the similarity between healthy and treatment-effective groups, as well as pretreatment and treatment-invalid groups, was close. Statistical analysis showed that there were differences in the diversity index and richness among the healthy, treatment-effective, pretreatment and treatment-invalid groups. The abundance of *Klebsiella oxytoca* in the treatment-effective LPRD group was lower than that of the treatment-invalid LPRD group. The abundance of *Klebsiella oxytoca* can distinguish treatment-effective and -invalid groups (AUC = 0.859) with a sensitivity of 77.78% and specificity of 90.91%.

**Conclusion:**

There were differences in the diversity of cecal contents microbial community between treatment-invalid and treatment-effective LPRD groups. *Klebsiella oxytoca* has potential to distinguish treatment outcomes.

**Level of evidence:**

How common is the problem? Level 1. Is this diagnostic or monitoring test accurate? (Diagnosis) Level 4. What will happen if we do not add a therapy? (Prognosis) Level 5. Does this intervention help? (Treatment Benefits) Level 4. What are the COMMON harms? (Treatment Harms) Level 4. What are the RARE harms? (Treatment Harms) Level 4. Is this (early detection) test worthwhile?(Screening) Level 4.

## Highlights

• The laryngopharynx microbiota was different between the healthy and LPRD.
• The laryngopharynx microbiota between treatment-effective and -invalid LPRD.
• Klebsiella oxytoca has potential to distinguish treatment outcomes.

## Introduction

Laryngopharyngeal Reflux (LPR) refers to the phenomenon that gastric contents flow back to the upper esophageal sphincter, such as nasal cavity, oral cavity, pharynx, larynx, trachea, and lung.^1^ Due to the lack of protective mechanism for gastric acid and pepsin in the mucous membrane of the throat, LPR can cause mucosal surface damage in contact with gastric acid and cause LPR Disease (LPRD).^2^, ^3^ It is reported that about 10% of the patients visiting otolaryngology clinics have symptoms related to LPR, while half of the patients with hoarseness have symptoms attributed to LPR. If LPR left untreated on unrecognized, significant long-term complications commonly occur, such as chronic cough, recurrent laryngitis, oral cavity disorders/ulcers, and even subglottic stenosis.^4^ The association between LPR and laryngeal carcinoma seems to be emerging.^5^, ^6^ Lifestyle modifications, such as weight loss, stopping tobacco/alcohol use, and refraining from lying down within three hours after dinner, can reduce LPRD.^7^ Medications, including histamine-2 receptor antagonists and proton pump inhibitors, can help suppress acid production.^8^

During the study of LPRD pathology, the fact that the digestive tract is colonized by a large number of microorganisms has gradually been recognized as important.^9^ Bacteria can grow and colonize in the digestive tract, bringing many beneficial or unfavorable effects to the host.^10^ Intestinal bacteria can produce specific enzymes, which can not only ferment nutrients into absorbable forms, but also produce short-chain fatty acids with anti-inflammatory and immunomodulatory effects.^11^ For instance, *Klebsiella oxytoca* can activate MAVS pathways and the phosphorylation of Stat3 and ERK1/2, leading to kidney injury.^12^ The microbiota in the host homeostasis is important for the maintenance of the digestive mucosa and the mucosa recovery.^13^ Therefore, microbial communities deserve greater attention, especially that inhabit the nose, mouth, and throat.^10^ A report suggests that the development of LPRD may modify the laryngopharyngeal and oral microbiome, maintaining the mucosa and recovering impairments.^9^ A study on the alteration of the laryngopharyngeal microbiome in LPRD reveals the alteration is correlated with the occurrence, as well as development, of LRPD.^14^ However, the difference of the laryngopharyngeal microbiome among patients with different outcomes has only been explored superficially.

Therefore, we hypothesized that the laryngopharyngeal microbiota of the LPRD patient is different among the patients with different outcome to proton pump inhibitors. This current study would explore the difference of laryngopharyngeal microbiota species and abundance between LRPD patients and healthy people, as well as LPRD patients with different treatment outcomes.

## Methods

### Study design and patient selection

In this study, the case-control method was used to collect the adult patients who were diagnosed as LPRD according to the “Experts consensus on diagnosis and treatment of laryngopharyngeal reflux disease (2015)”^15^ from January 2021 to June 2022. The diagnostic criteria for LPRD were reflux symptom index > 13, or reflux finding score > 7, corroborated by either an esophagogastroduodenoscopy (erosive esophagitis or Barrett esophagus) or positive prolonged esophageal pH monitoring. The enrolled LPRD patients were designed as the LPRD group. At the same time, healthy volunteers were selected as the control group. All healthy volunteers were between 18 and 70 years old, with no LPRD symptoms, such as hoarseness, dysphagia, globus, regurgitation, heartburn, cough/throat clearing, or excessive throat mucus, within 2 months of the study. Individuals with a history of severe systemic diseases, laryngopharyngeal surgery, or hiatal hernia, were excluded from the control group. Those who had received any treatments for OSAHS or LPR were also excluded.The subjects were fully informed of the relevant information and signed the informed consent form. This study was designed in compliance with the Declaration of Helsinki and reviewed by ethical review committee of The Fourth Affiliated Hospital, Zhejiang University School of Medicine. Informed consent was obtained from all individual participants included in the study.

Inclusion criteria for PDRD patients: 1) The Reflux Symptom Index (RSI) was more than 13 and/or the Reflux Finding Score (RFS) was more than 7; 2) More than 3 laryngopharyngeal reflux events within 24 h (24 h multichannel intraluminal impedance-pH monitor); 3) No history of taking proton pump inhibitors or other drugs that inhibit gastric acid secretion within 1 month before the first visit; 4) No history of taking preparations of antibiotics, micro-ecology-related products, non-steroidal anti-inflammatory drugs, immunosuppressants, hormones and other drugs that affect the microbiota.

Exclusion criteria for all subjects: 1) Those with other organic diseases at the throat (such as benign or malignant tumors of the throat); 2) Those who have chronic systemic diseases and need long-term medication (such as hypertension, diabetes, kidney disease, etc.); 3) Patients who were previously diagnosed as gastroesophageal reflux disease; 4) Patients previously diagnosed as chronic sinusitis, chronic tonsillitis, allergic rhinitis or other allergic diseases; 5) Those who have upper respiratory tract infection; 6) Those who take drugs for pharyngitis in the past month; 7) Those with mental disorders or pregnancy.

### Treatment

All enrolled patients were given Esomeprazole Magnesium Enteric-coated Tablets (AstraZeneca AB) 20 mg, twice a day, 30–60 min before meals, for 8 weeks. Follow-up was conducted at the 8th week of treatment.

### Treatment effectiveness evaluation

The curative effect was evaluated after treatment, and the RSI/RFS scale was collected by the researcher the same as before medication.

Effective: symptoms improved by more than 50%, RSI less than 13 or decreased by more than 4.

Invalid: no improvement in symptoms, and no reduction in RSI.

### Secretion collection

Under the electronic laryngoscope, the secretion from the laryngopharynx (piriform sinus) were taken from patients in fasting state, into sterile culture tubes and sterile Eppendorf tubes. Sterile culture tubes were sent to the laboratory within 1 h for microbial culture. The sterile Eppendorf tubes were quickly frozen by liquid nitrogen and stored at −80°.

### Microbial culture method

Pharyngeal swabs were placed on blood agar plates, chocolate agar plates, and China Blue agar plates and incubated at 37 °C ± 1 in atmosphere of air or 5% CO_2_. After incubation, bacterial identification was performed on an automatic Phoenix system (Becton Dickinson, USA) or an automatic Vitek2 system (bioMérieux Inc., France).

### Preparation of DNA sample

Total nucleic acid isolation was conducted in duplicate using the MagMAX Microbiome Ultra Nucleic Acid Isolation Kit (Applied Biosystems, USA). Yields were evaluated ed on a NanoDrop™ 2000 Spectrophotometer (Thermo Scientific, USA).

### PCR amplification

The diluted DNA sample (1 ng/μL) was used as the template to complete PCR amplification targeting the hypervariable region of the 16S rRNA region of bacteria using the primers.^16^ PCR products were purified and recovered using Poly-Gel DNA Extraction Kit (Solarbio, China).

### Denaturing gradient gel electrophoresis (DGGE) of amplified fragments

PCR products (10 μL) were analyzed by DGGE. Under the condition of 150 V and 60 °C, the PCR product was denatured by polyacrylamide gel at a gradient of 35%‒55% and a mass fraction of 8% in 1 × TAE buffer for 5 h. After DGGE, deionized water was poured out. The gels were fixed and stained with s silver dye solution. The differentiation analysis of the DGGE gel atlas was carried out with Quantity One software, and the allowable error was 2%, to collect the number of bands and gray values. The Dice index calculated by the Quantity One software was used to construct the system tree for cluster analysis between different bacterial groups. Shannon-Wiener index and evenness index represented the diversity and evenness.

### PCR for quantification of total bacteria and *Klebsiella oxytoca*

PCR products was reversely transcribed into cDNA with OneScript Hot cDNA Synthesis Kit (Abcam) using random primers. For total bacterial DNA quantification, droplet digital PCR reaction was prepared with sample DNA, EvaGreen Supermix (BioRad, USA), and primers. For quantification of *Klebsiella oxytoca*, Klebsiella oxytoca PCR kit was used. Samples were placed into a QX200 Automated Droplet Generator (BioRad, USA) and subjected to PCR amplification. Amplification product was placed in a QX200 Droplet Reader (BioRad, USA), to space out positive and negative droplets based on their individual fluorescence.

### Statistical analysis

According to the previous literature, we estimated that the RSI score of the LPRD group would be 4.5 points higher than that of the control group after treatment.^17^ A sample size of 29 patients per group was calculated with an assumption of alpha value of 0.05% and 80% power using a two-tailed *t*-test. Considering a dropout rate of 10%, 33 patients will be recruited for each group. In actual recruitment, we will plan to recruit 35 participants for the intervention and control groups. We will plan to recruit sufficient participants by posting posters in ENT clinics for 4-months.

Statistical analyses were completed in GraphPad Prism 9 and SPSS 22.0 statistical software. Shapiro-Wilk test was used to check normal distribution for all data sets. For non-normally distributed data, log-transformed or non-parametric tests were used to determine the statistical significances. For normally or lognormally distributed data, unpaired two-tailed Student’s *t*-test or one-way analysis of variance was used to determine the statistical significances. The Pearson Chi-Square test was used for comparing categorical data between treatment effective and invalid groups. The predictive performance of *Klebsiella oxytoca* was assessed by Receiver Operating Characteristic (ROC) curve analysis. A *p*-value < 0.05 refers to a statistical significance.

## Results

### Basic characteristics of the subjects

A total of 57 subjects were finally included in the study, of which 29 were in the LPRD group and 28 in healthy group (with one dropped out). LPRD group included 18 males and 11 females, with an average age of 49.52 ± 7.90 years. The healthy group included 16 males and 12 females, with an average age of 48.64 ± 8.76 years. There was no significant difference in age, gender, Body Mass Index (BMI), smoking history, drinking history, and history of gastropathy between the case group and the control group (Table 1). After treatment, the effective cases were 18 and invalid ones were 11 among patients with LPRD. There was no significant difference in age, gender, Body Mass Index (BMI), smoking history, drinking history, and history of gastropathy between the treatment-effective group and the treatment-invalid group (Table 2).

**Table 1:** Comparison of baseline characteristic between healthy and Laryngopharyngeal Reflux Disease (LPRD) group.

Parameters	Health (n = 28)	LPRD (n = 29)	*p*-values
Age (years)	48.64 ± 8.76	49.52 ± 7.90	0.693
Male/female (n/n)	16/12	18/11	0.705
BMI	23.02 ± 2.71	22.65 ± 2.45	0.592
Smoking history (n)	16	18	0.705
Drinking history (n)	12	14	0.681
History of gastropathy	9	9	0.928

BMI, Body Mass Index.

**Table 2:** Comparison of baseline characteristic between treatment-effective and -invalid Laryngopharyngeal Reflux Disease (LPRD) group.

Parameters	Invalid (n = 11)	Effective (n = 18)	*p-*values
Age (years)	51.91 ± 8.29	48.06 ± 7.50	0.208
Male/female (n/n)	8/3	10/8	0.355
BMI	21.92 ± 1.55	23.10 ± 2.81	0.213
Smoking history (n)	7	11	0.892
Drinking history (n)	5	9	0.812
History of gastropathy	3	6	0.732
RSI	19.82 ± 4.40	20.17 ± 4.55	0.841
RFS	11.82 ± 3.37	10.11 ± 2.76	0.149

BMI, Body Mass Index; RSI, Reflux Symptom Index; RFS, Reflux Finding Score.

### Microbial culture results

**Figure 1:**
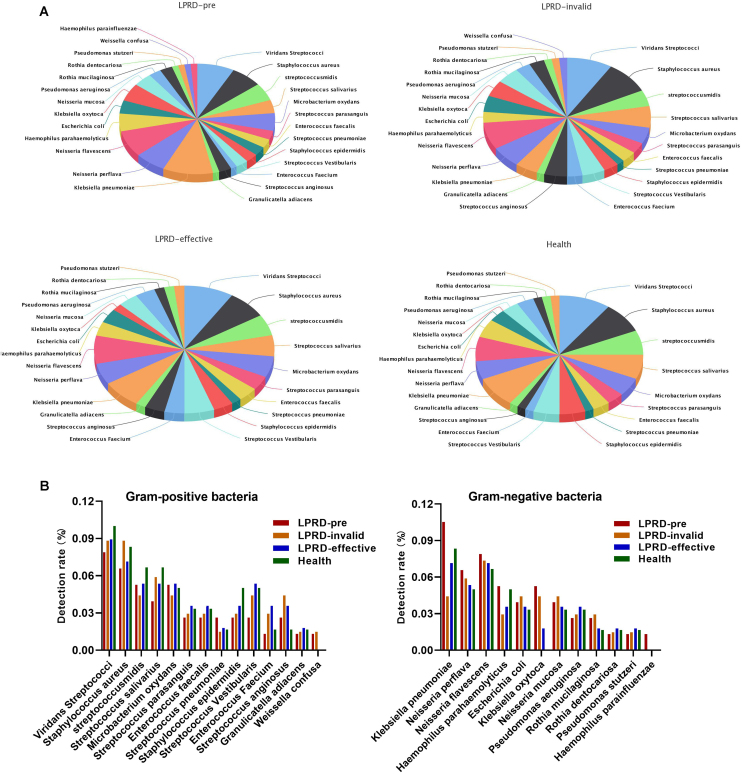
Composition of microbiome by bacterial culture method. (A) Pie charts of microbiome microbiome composition. (B) The distribution of Gram-positive and negative bacteria.

A total of 86 specimens of throat secretions were cultured in this study. As shown in Fig. 1A, the bacteria in healthy participants were 26 kinds (60 strains). Among the 29 cases of LPRD pretreatment, 28 kinds of bacteria and 77 strains were cultivated, including 14 kinds of gram-positive bacteria and 12 kinds of gram-negative bacteria. The top three bacteria were Klebsiella pneumoniae, Viridans Streptococci, and Neisseria flavescens. After treatment, the effective cases were cultured to 26 kinds of bacteria, while the invalid ones were cultured to 27 kinds of bacteria. The detection rate of *Klebsiella oxytoca* and Klebsiella pneumoniae was different among the four groups (Fig. 1B).

### Microbial diversity in patients with LPRD and healthy controls

**Figure 2:**
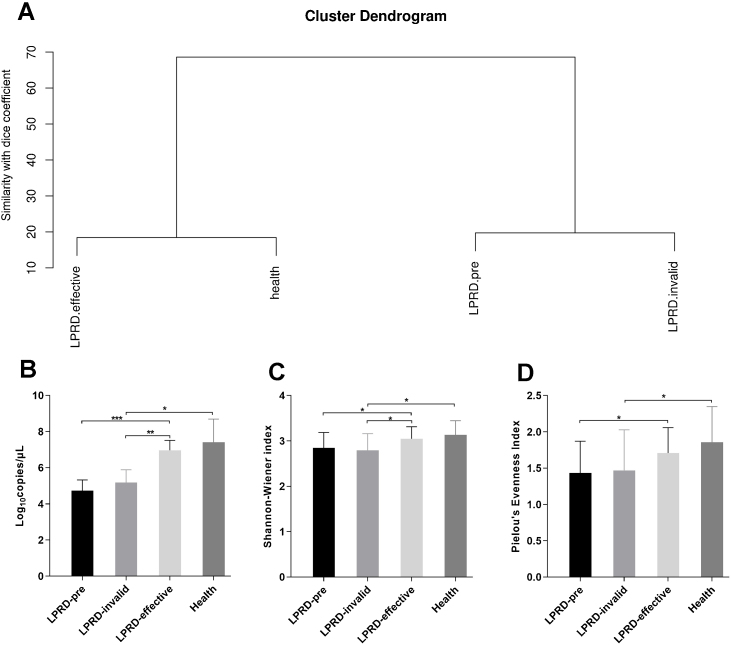
Comparisons of alpha diversity indices among the groups. (A) Dendrogram constructed based on Dice coefficients derived from DGGE fingerprints. (B) Total bacterial DNA copies were quantified by droplet digital PCR. The microbial alpha diversity within each sample was analyzed based on the Shannon-Wienner diversity index (C) and the Pielou's evenness (D).

According to the intensity and migration degree of the bands in DGGE, the similarity between samples is calculated according to the dice coefficient. The cluster analysis was conducted and the generated system tree. As shown in Fig. 2A, the cluster similarity between the health group and the treatment-effective group was high, as well as the similarity between the LPRD-pretreatment and treatment-invalid group. Droplet digital PCR reaction quantified the total bacteria (Fig. 2B), showing that total bacteria number in treatment-effective group was significantly higher than that in the pre-treatment group and treatment-invalid group. This increase in bacteria abundance was accompanied by a rise in bacterial alpha diversity as measured by their Shannon Index (Fig. 2C) and evenness index (Fig. 2D).

### Correlation of *Klebsiella oxytoca* with treatment outcome

**Figure 3:**
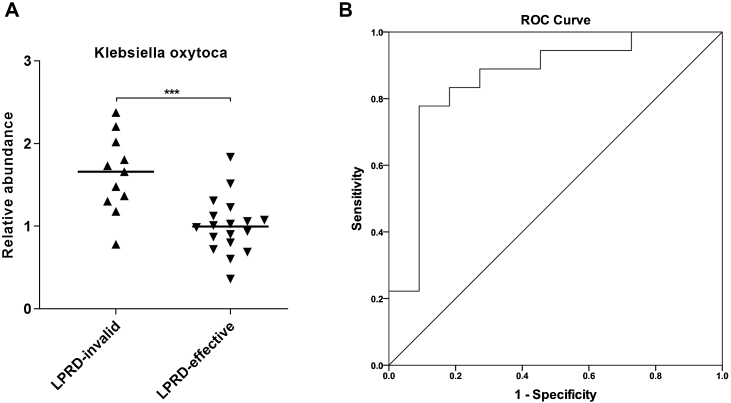
*Klebsiella oxytoca* had potential to discriminate the different outcomes of LPRD patients. (A) Relative copies of *Klebsiella oxytoca* were quantified by droplet digital PCR. (B) The ROC curve was constructed based on the relative copies of *Klebsiella oxytoca*.

Next, *Klebsiella oxytoca* aroused our interest. By using droplet digital PCR, we quantified *Klebsiella oxytoca* in the treatment-effective and -invalid groups. As shown in Fig. 3A, compared with the treatment-effective group, the treatment-invalid group showed lower *Klebsiella oxytoca* abundance. Then, the ROC curve was plotted to assess the discriminatory ability of *Klebsiella oxytoca* abundance in treatment outcomes. As shown in Fig. 3B, the abundance of *Klebsiella oxytoca* was an effective biomarker to distinguish treatment-effective and -invalid groups (AUC = 0.859) with a sensitivity of 77.78% and specificity of 90.91%.

## Discussion

According to the microecology theory, microorganisms and their growing environment together constitute the microecology environment.^18^ The balance between microorganisms and host is closely related to the occurrence and development of host diseases.^19^ In the long-term evolution process, some specific normal flora that forms an ecosystem and maintains relative balance is formed in the lumen connecting the biological host and the outside world.^20^ In general, the microbiota maintains a dynamic balance in its growth environment, and changes in its species or density can promote the transformation of host health and disease status.^21^ The interaction of host, microorganism and environment sometimes changes the balance state of microecology from physiological combination state to pathological combination state, resulting in the imbalance of microecology.^22^ Any microorganism that exceeds a certain amount or migrates to other parts for colonization may lead to host disease. The throat is the channel connecting the mouth, upper and lower respiratory tract and esophagus. It is connected with the external environment through the mouth and nasal cavity. Many microorganisms are distributed in this area.^23^ This experiment took LPRD patients as the research objects, and analyzed the difference of microbial community in pharyngeal swabs from LPRD and normal people. After microbial culture and DGGE detection, it was found that the bacterial composition of LPRD treatment-effective group was similar to that of healthy patients. The bacterial composition of the ineffective group was similar to that before treatment. In this experiment, Shannon-Weaver index was also used to analyze the microbial community of mice in the diabetes group and the healthy control group, indicating that the microbial diversity and richness in the throat swabs of LPRD patients before treatment and treatment-ineffective LPRD patients were reduced. Moreover, the abundance of Klebsiella acidogenesis can effectively distinguish the treatment effective group from the treatment ineffective group.

The composition of microbiota mainly depends on the host and the environmental factors in and out of the host, such as nutrition source, mucosal structure and host immunity.^19^, ^23^ In the current study, the diversity of pharyngeal microbiome in patients with LPRD is lower than healthy controls. This is in line with a report from Chen et al., which revealed a distinct microbiota dysbiosis in patients with LPRD and healthy individuals.^14^ Moreover, it has been revealed that the diversity of gut microbiome in patients with reflux disease can influence the gut microbiome of patients.^24^ It has been reported that alterations of both the gut and throat microbiota happen after Helicobacter pylori eradication therapy containing proton pump inhibitor, whereas the throat bacterial alteration appeares to persist.^25^ Notably, we found a distinct microbiota abundance distribution between treatment-effective and -invalid groups. This paved a way for the further study of the proton-pump-inhibitor-resistant bacteria.

In addition, this study found that the abundance of *Klebsiella oxytoca* was significantly increased in the treatment-ineffective group, which is a good biomarker to distinguish patients with different outcomes. Klebsiella are usually resistant to multiple antibiotics, and serve as significant human pathogens.^26^ *Klebsiella oxytoca*, a Gram-negative bacterium, belongs to the genus Klebsiella within the family Enterobacteriaceae.^27^ In humans, *Klebsiella oxytoca* can be found in gut microflora, the stool, the skin and in the oropharynx. Largely masked by the notorious-relative Klebsiella pneumoniae, *Klebsiella oxytoca* is relatively under the radar,^28^ despite its importance in causing various infections as an important human pathogen.^29^ Recently, oral Klebsiella was discovered to induce dysbiosis and inflammation in ectopic colonization of the gut, bringing a hypothesis that the oral cavity provides a reservoir for Klebsiella.^30^ A previous study observed oral *Klebsiella oxytoca* appeared to be transcriptionally active and recoverable after the death phase throughout the ecological perturbation of long-term starvation.^31^ This implies the pathogenicity of *Klebsiella oxytoca* in oral cavity. Further, we discovered that *Klebsiella oxytoca* may serve as a useful biomarker for LPRD outcomes based on the ROC analysis. However, the reliability of *Klebsiella oxytoca* as a biomarker needs further exploration in large-scale prospective research.

## Conclusion

In conclusion, the laryngopharyngeal microbiome composition in pre-treatment, treatment-effective, and treatment-invalid LPRD patients was similar to that of healthy participants, whereas differences were found in the dominant flora. This study suggested that *Klebsiella oxytoca* may serve as a useful biomarker for LPRD outcomes. Therefore, our understanding of LPRD and laryngopharyngeal microbiota may play a role in LPRD therapy.

## Funding

This study was funded by Science and Technology program of Yiwu Science and Technology Bureau (Grant No. 20-3-071).

## Conflicts of interest

The authors declare no conflicts of interest.

## References

[1] Barrett C.M., Patel D., Vaezi M.F. (2020). Laryngopharyngeal reflux and atypical gastroesophageal reflux disease. Gastrointest Endosc Clin N Am..

[2] Lechien J.R., Saussez S., Karkos P.D. (2018). Laryngopharyngeal reflux disease: clinical presentation, diagnosis and therapeutic challenges in 2018. Curr Opin Otolaryngol Head Neck Surg..

[3] Kowalik K., Krzeski A. (2017). The role of pepsin in the laryngopharyngeal reflux. Otolaryngol Pol..

[4] Lechien J.R., Hans S., Calvo-Henriquez C., Baudouin R., Saussez S. (2022). Laryngopharyngeal reflux may be acute, recurrent or chronic disease: preliminary observations. Eur Arch Otorhinolaryngol..

[5] Han H., Lyu Q., Zhao J. (2022). Laryngopharyngeal reflux in hypertrophic laryngeal diseases. Ear Nose Throat J..

[6] Jing W., Luo W., Lou L. (2023). Diagnostic utility of salivary pepsin in laryngopharyngeal reflux: a systematic review and meta-analysis. Braz J Otorhinolaryngol..

[7] Saniasiaya J., Kulasegarah J. (2023). The link between airway reflux and non-acid reflux in children: a review. Braz J Otorhinolaryngol..

[8] Lechien J.R., Mouawad F., Bobin F., Bartaire E., Crevier-Buchman L., Saussez S. (2021). Review of management of laryngopharyngeal reflux disease. Eur Ann Otorhinolaryngol Head Neck Dis..

[9] Lechien J.R., De Vos N., Everard A., Saussez S. (2021). Laryngopharyngeal reflux: the microbiota theory. Medical Hypotheses..

[10] Proctor D.M., Relman D.A. (2017). The landscape ecology and microbiota of the human nose, mouth, and throat. Cell Host Microbe..

[11] Cani P.D., Van Hul M., Lefort C., Depommier C., Rastelli M., Everard A. (2019). Microbial regulation of organismal energy homeostasis. Nat Metab..

[12] Linh H.T., Iwata Y., Senda Y., Sakai-Takemori Y., Nakade Y., Oshima M. (2022). Intestinal bacterial translocation contributes to diabetic kidney disease. J Am Soc Nephrol..

[13] Xu X., Wang Z., Zhang X. (2015). The human microbiota associated with overall health. Crit Rev Biotechnol..

[14] Chen H., Wang H., Yang F., Wang M., Chen X. (2022). Distinct microbiota dysbiosis in patients with laryngopharynx reflux disease compared to healthy controls. Eur Arch Otorhinolaryngol..

[15] Subspecialty Group of Laryngopharyngology EBoCJoOH, Zhi NSJZEBYHTJWKZ. [Experts consensus on diagnosis and treatment of laryngopharyngeal reflux disease (2015)]. 2016;51:324-6. 10.3760/cma.j.issn.1673-0860.2016.05.002 27220289

[16] Dar S.A., Stams A.J., Kuenen J.G., Muyzer G. (2007). Co-existence of physiologically similar sulfate-reducing bacteria in a full-scale sulfidogenic bioreactor fed with a single organic electron donor. Appl Microbiol Biotechnol..

[17] Shen H., Han Y., Wu D., Hu L., Ma Y., Wu F. (2022). Trial of transcutaneous electrical acupoint stimulation in laryngopharyngeal reflux disease: study protocol for a randomized controlled trial. Trials..

[18] Cappellato M., Baruzzo G., Patuzzi I., Di Camillo B. (2021). Modeling microbial community networks: methods and tools. Curr Genomics..

[19] Dong C., Li D., Wang Z., Bao Z. (2021). Oral microbial diversity formed and maintained through decomposition product feedback regulation and delayed responses. Evid Based Complement Alternat Med..

[20] Huang K., Wu L., Yang Y. (2021). Gut microbiota: an emerging biological diagnostic and treatment approach for gastrointestinal diseases. JGH Open..

[21] Wu Y., Wang C.Z., Wan J.Y., Yao H., Yuan C.S. (2021). Dissecting the interplay mechanism between epigenetics and gut microbiota: health maintenance and disease prevention. Int J Mol Sci..

[22] Fekete S., Szabó D., Tamás L., Polony G. (2019). The role of the microbiome in otorhinolaryngology. Orv Hetil..

[23] Domènech L., Willis J., Alemany-Navarro M., Morell M., Real E., Escaramís G. (2022). Changes in the stool and oropharyngeal microbiome in obsessive-compulsive disorder. Sci Rep..

[24] Flynn M., Dooley J. (2021). The microbiome of the nasopharynx. J Med Microbiol..

[25] Wang Z.-J., Chen X.-F., Zhang Z.-X., Li Y.-C., Deng J., Tu J. (2017). Effects of anti-Helicobacter pylori concomitant therapy and probiotic supplementation on the throat and gut microbiota in humans. Microb Pathog..

[26] Leitner E., Zarfel G., Luxner J., Herzog K., Pekard-Amenitsch S., Hoenigl M. (2015). Contaminated handwashing sinks as the source of a clonal outbreak of KPC-2-producing Klebsiella oxytoca on a hematology ward. Antimicrob Agents Chemother..

[27] Yang J., Long H., Hu Y., Feng Y., McNally A., Zong Z. (2022). Klebsiella oxytoca complex: update on taxonomy, antimicrobial resistance, and virulence. Clin Microbiol Rev..

[28] Moradigaravand D., Martin V., Peacock S.J., Parkhill J. (2017). Population structure of multidrug resistant Klebsiella oxytoca within hospitals across the UK and Ireland identifies sharing of virulence and resistance genes with K. pneumoniae. Genome Biol Evol..

[29] Neog N., Phukan U., Puzari M., Sharma M., Chetia P. (2021). Klebsiella oxytoca and emerging nosocomial infections. Curr Microbiol..

[30] Atarashi K., Suda W., Luo C., Kawaguchi T., Motoo I., Narushima S. (2017). Ectopic colonization of oral bacteria in the intestine drives T(H)1 cell induction and inflammation. Science..

[31] Baker J.L., Hendrickson E.L., Tang X., Lux R., He X., Edlund A. (2019). Klebsiella and Providencia emerge as lone survivors following long-term starvation of oral microbiota. Proc Natl Acad Sci U S A..

